# Non enzymatic upregulation of tissue factor expression by gamma-glutamyl transferase in human peripheral blood mononuclear cells

**DOI:** 10.1186/s12959-016-0119-8

**Published:** 2016-11-04

**Authors:** Valentina Scalise, Cristina Balia, Silvana Cianchetti, Tommaso Neri, Vittoria Carnicelli, Riccardo Zucchi, Maria Franzini, Alessandro Corti, Aldo Paolicchi, Alessandro Celi, Roberto Pedrinelli

**Affiliations:** 1 Dipartimento di Patologia Chirurgica, Medica, Molecolare e dell’Area Critica, Università di Pisa, Pisa, Italy; 2 Dipartimento di Ricerca Traslazionale e delle Nuove Tecnologie in Medicina e Chirurgia, Università di Pisa, Pisa, Italy

**Keywords:** Gamma-glutamyltransferase, Tissue Factor, Cytokines, Oxidative Stress, NFkB

## Abstract

**Background:**

Besides maintaining intracellular glutathione stores, gamma-glutamyltransferase(GGT) generates reactive oxygen species and activates NFkB, a redox-sensitive transcription factor key in the induction of Tissue Factor (TF) gene expression, the principal initiator of the clotting cascade. Thus, GGT might be involved in TF-mediated coagulation processes, an assumption untested insofar.

**Methods:**

Experiments were run with either equine, enzymatically active GGT or human recombinant (hr) GGT, a wheat germ-derived protein enzymatically inert because of missing post-translational glycosylation. TF Procoagulant Activity (PCA, one-stage clotting assay), TF antigen(ELISA) and TFmRNA(real-time PCR) were assessed in unpooled human peripheral blood mononuclear cell(PBMC) suspensions obtained from healthy donors through discontinuous Ficoll/Hystopaque density gradient.

**Results:**

Equine GGT increased PCA, an effect insensitive to GGT inhibition by acivicin suggesting mechanisms independent of its enzymatic activity, a possibility confirmed by the maintained stimulation in response to hrGGT, an enzymatically inactive molecule. Endotoxin(LPS) contamination of GGT preparations was excluded by heat inactivation studies and direct determination(LAL method) of LPS concentrations <0.1 ng/mL practically devoid of procoagulant effect. Inhibition by anti-GGT antibodies corroborated that conclusion. Upregulation by hrGGT of TF antigen and mRNA and its downregulation by BAY-11-7082, a NFkB inhibitor, and N-acetyl-L-cysteine, an antioxidant, was consistent with a NFkB-driven, redox-sensitive transcriptional site of action.

**Conclusions:**

GGT upregulates TF expression independent of its enzymatic activity, a cytokine-like behaviour mediated by NFκB activation, a mechanism contributing to promote acute thrombotic events, a possibility in need, however, of further evaluation.

## Background

Gamma-glutamyltransferase [GGT; (5-l-glutamyl)-peptide⁄amino acid 5-glutamyl transferase; EC 2.3.2.2], a member of the structural superfamily of the N-terminal nucleophilic hydrolases expressed by a wide number of cell types [1] including circulating monocytes [2, 3], hydrolyzes extracellular glutathione (GSH) to provide cysteine for its intracellular re-synthesis. Along with its pivotal role in that antioxidant biological process [1], however, GGT also generates reactive oxygen species (ROS) [4] and activates NFkB [5, 6], a redox-sensitive transcription factor [7] key in Tissue Factor (TF) gene expression [8], a major regulator of haemostasis and thrombosis [8, 9]. Therefore, deductive reasoning makes it plausible to hypothesize a cross-talk between GGT and TF, a possibility consistent with the highly consistent epidemiological association of circulating GGT levels with acute coronary events (see [10] for a review), a pathological process favoured by TF (see [11] for a review). The assumption, corroborated by recent reports of enzymatically active protein in human atheromatous plaques [12], requires, however, to document a mechanistic link between GGT and TF for which at the moment no evidence is available.

## Aims

On the basis of that background, our aim was to evaluate the relationship between GGT and TF expression in human peripheral blood mononuclear cell (PBMC)s, a cell preparation capable of rapid TF induction in response to various proinflammatory stimuli [8].

## Methods

### Chemicals and standards

Unless stated otherwise, all reagents were from Sigma, Milano, Italy.

### Cell isolation and culture

Human PBMC suspensions were obtained from unpooled buffy coats left over from blood bank draws taken from healthy donors with the approval of the local ethics committee of the Azienda Ospedaliero Universitaria Pisana, kept at room temperature and utilized within a maximum of 4 h from withdrawal. As detailed elsewhere [13], leukocytes were isolated from fresh buffy coats diluted 1:1 with sodium citrate 0.38 % in saline solution, mixed gently with 0.5 volume of 2 % Dextran T500 and left for 40 min for erythrocyte sedimentation. The leukocyte-rich supernatant was recovered and centrifuged for 10 min at 200xg. The pellet was resuspended in 30 mL of sodium citrate solution, layered over 15 mL of Ficoll-Hypaque and centrifuged for 30 min at 400xg at 20 °C. The PBMC-rich ring (3×10^6^ cells/mL) was recovered, washed twice in sodium citrate 0.38 % and resuspended in polypropylene tubes in no glucose RPMI 1640 medium supplemented with 100 U/mL penicillin-streptomycin.

All reagents and solutions used for cell isolation and cultures were prepared with endotoxin-free water and glassware was rendered endotoxin-free by exposure to high temperature. Drugs were kept in stock solution and diluted in serum-free RPMI at the appropriate concentrations immediately before use. Cell viability, as assessed by dimethyl thiazolyl diphenyl tetrazolium (MTT), was verified (>85 % of viable cells) throughout all experimental phases.

The final PBMC preparations typically contain 25–35 % monocytes, negligible proportions of neutrophils (<5 %) and 65–75 % lymphocytes, a cell line with some but limited procoagulant potential [14]. Moreover, interferences from contaminating platelets [15] were excluded by absent procoagulant activity in clotting assays carried out in PBMC-free preparations as verified in pilot experiments, so that stimulated TF expression should be considered by and large a result of activated monocytes.

### GGT

**Fig. 1:**
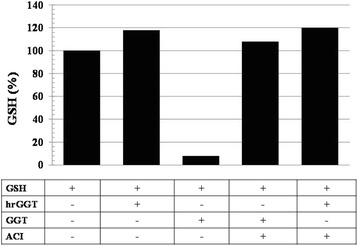
Absent hydrolytic effect of hrGGT (0.5 μg/mL) in contrast with the complete GSH cleavage obtained by equine GGT (100 mU/mL) reversed by acivicin (ACI, 10^−4^M), an irreversible GGT inhibitor [20] inactive on hrGGT. Results from a single experimental set after overnight GSH incubation and expressed as percent changes from control. GSH was measured according to standard methods [19]

GGT experiments were carried out by using either equine, enzymatically active GGT purified according to already described procedures [16], or human recombinant(hr)GGT (Abnova, Taipei, Taiwan), a protein synthesized by wheat germ eukaryotic translational apparatuses in which lack of post-translational glycosylation forbids the generation of enzymatically active molecules [17, 18]. Confirming that assumption, GSH as measured by standard methods [19] was not cleaved by hrGGT in contrast with the hydrolysis induced by equine GGT, an effect this latter abolished by acivicin (10^−4^ M), a glutamate analogue with potent, non competitive GGT inhibiting properties [20], inactive on hrGGT (Fig. 1).

To exclude interferences from endotoxin (LPS) contamination [21], experiments were carried out with GGT preparations boiled for 30 min before their addition to PBMCs, a procedure expected to abrogate the biological effect of GGT but not the procoagulant power of heat-resistant LPS [21]. In the light of heat sensitivity of some LPS strains [22], endotoxin concentration was also measured in hrGGT-primed cultures by LAL (Limulus Amebocyte Lysate) chromogenic endpoint assay (Hycult Biotech, Uden, The Netherlands) [23] and the procoagulant effect of contaminant LPS levels was then tested in quiescent PBMCs. Finally, we assessed the effect of hrGGT-inhibition by an anti-GGT polyclonal antibody (Abnova, Taipei, Taiwan), 2.5 μg/mL, a concentration chosen according to preliminary concentration-response experiments (not shown), as compared with the appropriate isotype IgG control.

The role of NFkB was indirectly assessed by using BAY-11-7082 (10^−5^M), a validated NFkB inhibitor [24], as well as N-acetyl-L-cysteine (NAC, 10^−3^M), an antioxidant with inhibitory effect on NFkB [25].

### Experimental methods

#### *TF Procoagulant Activity (PCA)*

PCA was assessed by a one-stage clotting time test in PBMCs disrupted by three freeze–thaw cycles as reported previously [26]. Disrupted cells (100 μL) were mixed with 100 μL of normal human plasma at 37 °C, adding 100 μl of 25 mM CaCl_2_ at 37 °C. Time to clot formation was recorded and values converted to arbitrary units (AU) by comparison with a standard human brain TF calibration curve covering clotting times from 20 to 600 s. The standard TF preparation was arbitrarily assigned a value of 1000 AU/mL and a representative conversion of clotting times to AU is as follows: 100 AU-21 s, 10 AU-40s, 1 AU-82 s, 0.1-187 s, 0.01 AU-375 s, 0.001 AU-600 s.

“Baseline” values were defined as clotting times above 375 s (0.01 AU) and refer to quiescent, non activated, untreated PBMCs. Experiments were run in triplicate and averaged.

#### *TF antigen(ag)*

Cells were disrupted by three repeated freeze–thaw cycles and debris pelleted by centrifugation at 100xg for 1 h at 4 °C and supernatants used for ELISA according to manufacturer’s instructions (Imubind TF kit Sekisui Diagnostics, West Malling, United Kingdom). Within and between assay variability was 3.5 and 5.5 %, respectively.

#### *TF mRNA*

Total RNA was extracted from PBMCs using the RNeasy mini kit (Qiagen, Hilden, Germany). RNA concentration and purity were determined by optical density measurement via Nanodrop (Thermo Fisher Scientific, Wilmington, Delaware USA). A mixture of 0.5 ng total RNA per sample was retro-transcribed with random primer-oligodT into complementary DNA (cDNA) using the Quantitect Reverse Transcription Kit (Qiagen, Hilden, Germany). The retro-transcription cycle was performed at 25 °C for 5 min, 42 °C for 30 min and 95 °C for 3 min. RealTime-PCR was carried out in a iQ5 Real Time PCR System and SsoAdvanced Sybr Green Supermix (Bio-Rad Laboratories, Hercules, CA) was employed on the basis of the manufacturer’s conditions: 95 °C, 30s; 40 cycles 95 °C, 5 s, 60 °C, 15 s;a final melting protocol with ramping from 65 °C to 95 °C with 0,5 °C increments of 5 s was performed. The primers sequence for RealTime-PCR were: TF, sense 5’-TTGGCAAGGACTTAATTTATACAC-3’, antisense 5’-CTGTTCGGGAGGGAATCAC-3’; GAPDH, sense: 5’-CCCTTCATTGACCTCAACTACATG-3’ and antisense: 5’-TGGGATTTCCATTGATGACAAGC-3’ (Invitrogen, Monza, Italy). All samples were analysed in duplicate and averaged. The relative expression of the target gene was normalized to the level of GAPDH in the same cDNA.

PCA and TFag were assayed after a 18 h incubation period while TFmRNA was evaluated after a 2 h interval. BAY 10–772, NAC and anti-GGT polyclonal antibody were added 30 min prior to GGT stimulation.

PCA, ELISA and Q-PCR results in each control and experimental group were all generated from suspensions containing equal number of cells (3×10^6^ PBMCs/mL).

### Statistics

Statistical differences were tested by Mann–Whitney test. Data were reported as means ± SD unless otherwise reported. A two-tailed p-level <0.05 was the threshold for statistical significance.

## Results

### GGT stimulates TF expression independent of its enzymatic properties

**Fig. 2:**
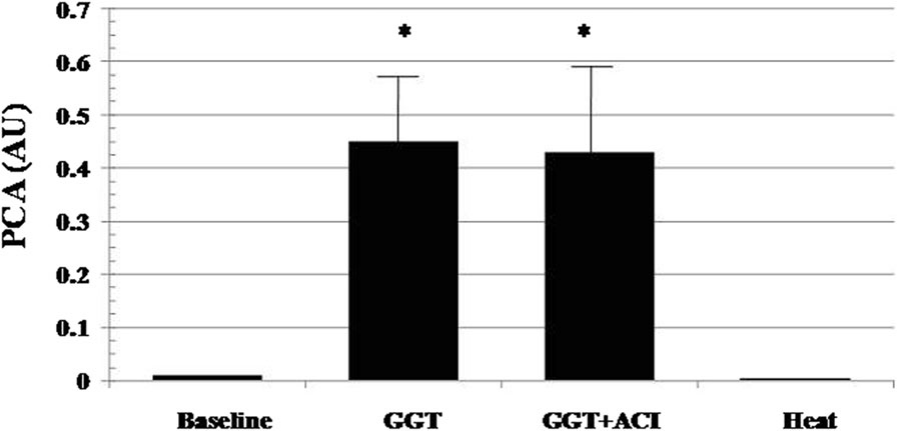
PCA stimulation by equine GGT (100 mU/mL), an effect insensitive to acivicin (ACI, 10^−4^), a GGT inhibitor [20], and abolished by heat inactivation of the enzyme. Means ± SD, *n* = 6, * *p* < 0.001 vs baseline. Baseline PCA refer to values ≤0.01 AU

Equine GGT (100 mU/mL) stimulated TF PCA, an effect insensitive to GGT inhibition by acivicin (10^−4^M) (Fig. 2). Incubation of the samples with an antihuman TF antibody (30 μg/mL; IgG, American Diagnostica) abolished more than 95 % of the PCA indicating its dependence upon TF (data not shown).

**Fig. 3:**
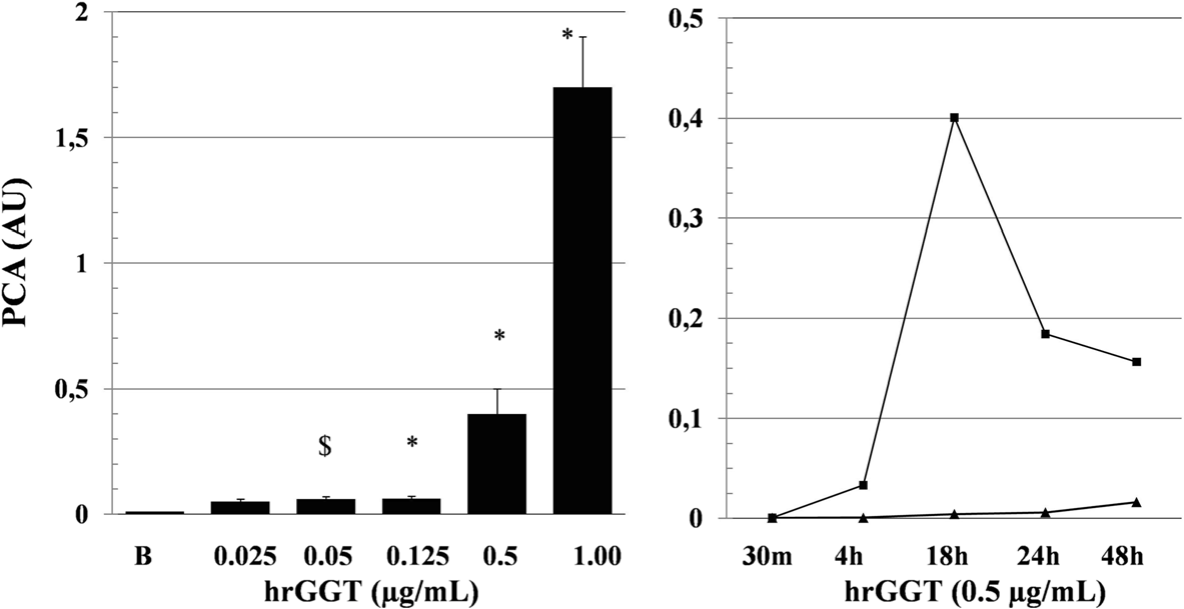
*Left panel*: Concentration-dependent increase in PCA in response to hrGGT, an enzymatically inactive protein. Means ± SD, *n* = 7, $ *p* < 0.05 and * *p* < 0.001 vs baseline (B). *Right panel*: Time course of PCA in PBMCs stimulated by hrGGT, 0.5 μg/mL (■), as compared to quiescent cell preparations (▲). Means, n = 4. Baseline PCA refer to values ≤0.01 AU

hrGGT, an enzymatically inactive protein induced a graded, concentration-dependent PCA stimulation without evidence of a plateau (Fig. 3, left panel). As a comparison, PCA in response to the peak hrGGT concentration (1.0 μg/mL) used in those studies was in the range obtained under identical experimental conditions by a well characterized procoagulant agonist such as LPS at a standard concentration of 0.1 μg/mL (from 0.01 ± 0.008 to 1.12 ± 0.2 AU) [13].

Taking into account its equieffectiveness to natural GGT, 100 mU/mL, all further studies were carried out with hrGGT, 0.5 μg/mL, and the time course of its effect on PCA is reported in Fig. 3, right panel.

### LPS contamination does not explain GGT-induced TF expression

Heat inactivation abrogated the effect of either natural GGT (Fig. 2) or hrGGT (data not shown).

**Fig. 4:**
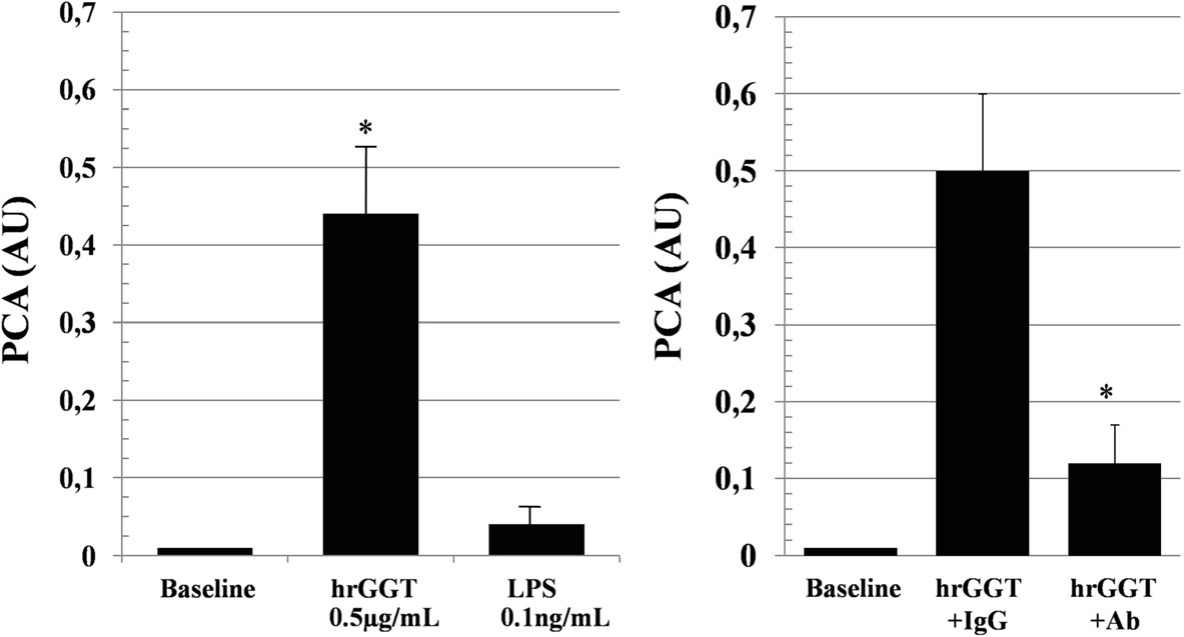
*Left panel*: Procoagulant effect of LPS concentrations in the contaminant range (0.1 ng/mL) relative to baseline values and hrGGT-primed values. Means ± SD, *n* = 3, *p < 0.001 vs LPS. *Right panel*: Inhibition of hrGGT(0.5 μg/mL)-stimulated PCA by a specific anti-hrGGT polyclonal IgG antibody (Ab, 2.5 μg/mL). Means ± SD, *n* = 7, *p < 0.001 vs hrGGT+ IgG isotype control. Baseline PCA refer to values ≤0.01 AU

LPS determination in hrGGT preparations averaged 0.07 ± 0.02 ng/mL (n = 4 replicates), a concentration that did not affect baseline PCA to a statistically significant extent quite in contrast with hrGGT (Fig. 4, left panel).

hrGGT-induced procoagulant responses were downregulated by an anti-GGT IgG polyclonal antibody (Fig. 4, right panel).

### hrGGT induces TF transcription by activating NFkB and increasing ROS generation

**Fig. 5:**
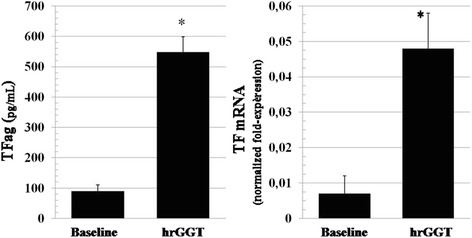
Stimulation by hrGGT (0.5 μg/mL) of TFag (*left panel*) and TFmRNA (*right panel*). Means ± SD, n = 13 and *n* = 9 each, *p < 0.001 vs baseline

**Fig. 6:**
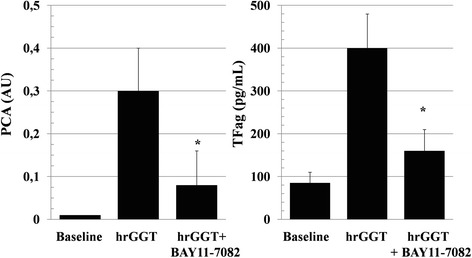
Inhibition of hrGGT (0.5 μg/mL)-stimulated PCA (*left panel*) and TFag (*right panel*) by BAY11-7082 (10^−5^M), a NFkB inhibitor [24]. Means ± SD, *n* = 7each, *p < 0.001 vs hrGGT. Baseline PCA refer to values ≤0.01 AU

hrGGT, 0.5 μg/mL, induced an increase in both TFag and mRNA (Fig. 5, left and right panel). BAY-11-7082 (10^−5^M) inhibited both GGT-stimulated TF protein and activity (Fig. 6, left and right panel), an inhibitory effect on PCA shared by NAC (10^−3^M, from 0.35 ± 0.07 to 0.09 ± 0.01, *p* < 0.001, *n* = 7).

## Discussion

Discussion of the effects of GGT on TF expression needs some preliminary comments about endotoxin contamination, an important concern in the light of the frequent pollution by that bacterial product of cell cultures even if grown in carefully controlled experimental conditions [22]. However, three orders of considerations make that possibility unlikely. First, GGT-induced PCA was abrogated by heat, a procedure generally ineffective on heat-resistant LPS [21]. Secondly, LPS levels in our preparation were less than <0.1 ng/mL, a concentration with a minor, if any, procoagulant action and certainly far less than that of GGT, a behaviour excluding an important role for heat-sensitive LPS strains [22]. Thirdly, inhibition of TF activity by an anti-GGT antibody provided additional proof of the specificity of the procoagulant response to hrGGT.

Having this background in mind, the main and original outcome of this study was the demonstration of the procoagulant properties of GGT and their independence from the enzymatic action of the molecule. That latter, quite intriguing conclusion is based upon the insensitivity of natural GGT-stimulated PCA to acivicin, a highly specific GGT inhibitor [20] and, more importantly, by the maintained procoagulant effect of hrGGT, a wheat germ-derived protein devoid of enzymatic activity because of a missing post-translational glycosylation apparatus [17, 18], an assumption fully verified by our GSH hydrolysis experiments reported in Fig. 1. Thus, hrGGT induced a concentration-dependent procoagulant effect, increased TFag and upregulated mRNA, a behaviour this latter showing that GGT-induced TF gene transcription is an early event since our mRNA assays were obtained after only 2 h of exposure to the molecule, quite similar in this regard to the response induced by unrelated cytokines and inflammatory agonists (e.g. [27]). However, additional studies are needed to evaluate the pattern of GGT-induced gene expression over a longer time course and the reader should also be aware that our PCR procedure based upon the use of GAPDH as a single and quite variable reference gene may present some technical limitations [28, 29]. Moreover, GGT-induced TF gene transcription was likely located at the level of NFkB activation given the inhibitory effect of BAY-10-772, a pharmacological antagonist [24] of that crucial controller of redox stimuli converging upon TF gene [7, 8]. The conclusion was strengthened by the negative modulation of PCA exerted by NAC, a sulfhydryl-group donor that, by increasing the antioxidant thiol pool and scavenging the excess of intracellular ROS, downregulates NFkB [25]. Therefore, our data, besides constituting, to the best of our knowledge, the first demonstration of a direct TF procoagulant effect of GGT, also provide consistent, albeit admittedly indirect, evidence for increased ROS production and NFkB activation as the pathophysiological mechanism linking GGT to TF expression in PBMCs.

That GGT might induce ROS generation is a concept dating back to two decades ago or so when Glass and Stark showed oxidative damage of cell surface proteins and membrane lipids as a consequence of GSH cleavage by GGT inducing auto-oxidation of the sulfur via a Fenton reaction resulting in the iron–dependent production of oxygen radicals [30]. However, that mechanism, which requires an enzymatically functional GGT molecule, cannot evidently apply to our data. Rather, direct, non enzymatic TF stimulation is closely consonant with similar results obtained in evaluating the role of the GGT as a bone resorbing factor [31]. The data open obvious questions about the GGT-operated signal transduction pathways upstream NFkB activation including the mechanisms allowing membrane permeation of the exogenous protein and the interaction with its intracellular targets. Although our results cannot answer this specific point, one might conjecture of a specific but insofar not identified GGT-receptor or lipid-driven endocytosis [32]. Perhaps, some role may play a chemokine-like CX3C motif contained in the GGT molecule [33] or exogenous GGT might mimick cytokine-like activities of GGT-related proteins produced by genes different from the GGT1 [34]. For example, the GGT2 gene located close to the GGT1 chromosomal region seems to encode for a full length, enzymatically inactive protein, not localized to the plasma membrane [35] and, according to some Authors, involved in redox modulation [36]. It might also be possible to envisage protein-protein interactions leading to the expression of other procoagulant cytokines. Additional unresolved questions to be addressed in future studies regard an understanding of how the enzymatic and non-enzymatic activity of GGT may complement each other as well as the role of circulating GGT levels in the activation of the TF procoagulant pathway.

The data reported in this study may have pathophysiological and clinical relevance. In fact, previous experiments have shown upregulated GGT transcription in response to NADPH oxidase-mediated ROS generation [37] as well as to agents endowed with TF-stimulating properties [8] such as Tumor Necrosis Factor(TNF)-alpha [38] and phorbol esters [39]. Moreover, monocytes, a cell line harbouring GGT [2, 3], have recently been shown to release a GGT fraction when exposed to LPS [40], a product of Gram-negative bacteria that initiates the pathogen-induced inflammatory response [41] of which activation of coagulation is a prominent component [9]. In that framework, thus, it is conceivable to hypothesize a vicious circle by which circulating or within-plaque [12, 40] GGT stimulates TF expression, and plaque-derived cytokines induce procoagulant GGT expression [38]. Both arms of this self-reverberating mechanism hinging around NFkB activation [42] may amplify the pro-thrombotic potential of vulnerable atheromatous plaques possibly in synergism with TF expressed by activated B- and T-lymphocytes [14, 43], a tempting but at the moment only speculative hypothesis worth being pursued in future studies.

## Conclusions

Our data provide the first evidence of a procoagulant action of GGT, a result that adds to the long [8] and enlarging (e.g. [44, 45]) list of TF-inducing agents acting through NFkB activation. In showing the independence of that effect from its enzymatic activity, the data raise the issue of the proinflammatory and prothrombotic potential of GGT as a cytokine-like protein. However, further work is needed to understand more precisely the extent to which in-vitro data can be transferred to in-vivo conditions and the possible pathophysiological importance of this mechanism in cardiovascular risk modulation.

## Abbreviations

ag: Antigen
AU: Arbitrary Units
CaCl_2_: Calcium Chloride
DNA: Deoxyribonucleic acid
ELISA: Enzyme-linked immunosorbent assay
GAPDH: Glyceraldehyde-3-phosphate dehydrogenase
GGT: Gamma-glutamyltransferase
GSH: Glutathione
Hr: Human recombinant
Ig: Immunoglobulin
LAL: Limulus Amebocyte Lysate
LPS: Lipopolysaccharide
MTT: Dimethyl thiazolyl diphenyl tetrazolium
NAC: N-acetyl-L-cysteine
NADP: Nicotinamide adenine dinucleotide phosphate
NFkB: Nuclear factor kappa-light-chain-enhancer of activated B cells
PBMC: Peripheral Blood Mononuclear Cell
PCA: Procoagulant activity
PCR: Polymerase Chain Reaction
RNA: Deoxyribonucleic acid
ROS: Reactive Oxygen Species
RPMI: Roswell Park Memorial Institute
SD: Standard deviation
TF: Tissue Factor
TNF: Tumour Necrosis Factor
